# Supplementary figures and images for: Identifying metabolic adaptations characteristic of cardiotoxicity using paired transcriptomics and metabolomics data integrated with a computational model of heart metabolism
Source: PLoS Comput Biol. 2024 Feb 29;20(2):e1011919. doi: 10.1371/journal.pcbi.1011919 (PMC10931521; doi:10.1371/journal.pcbi.1011919)

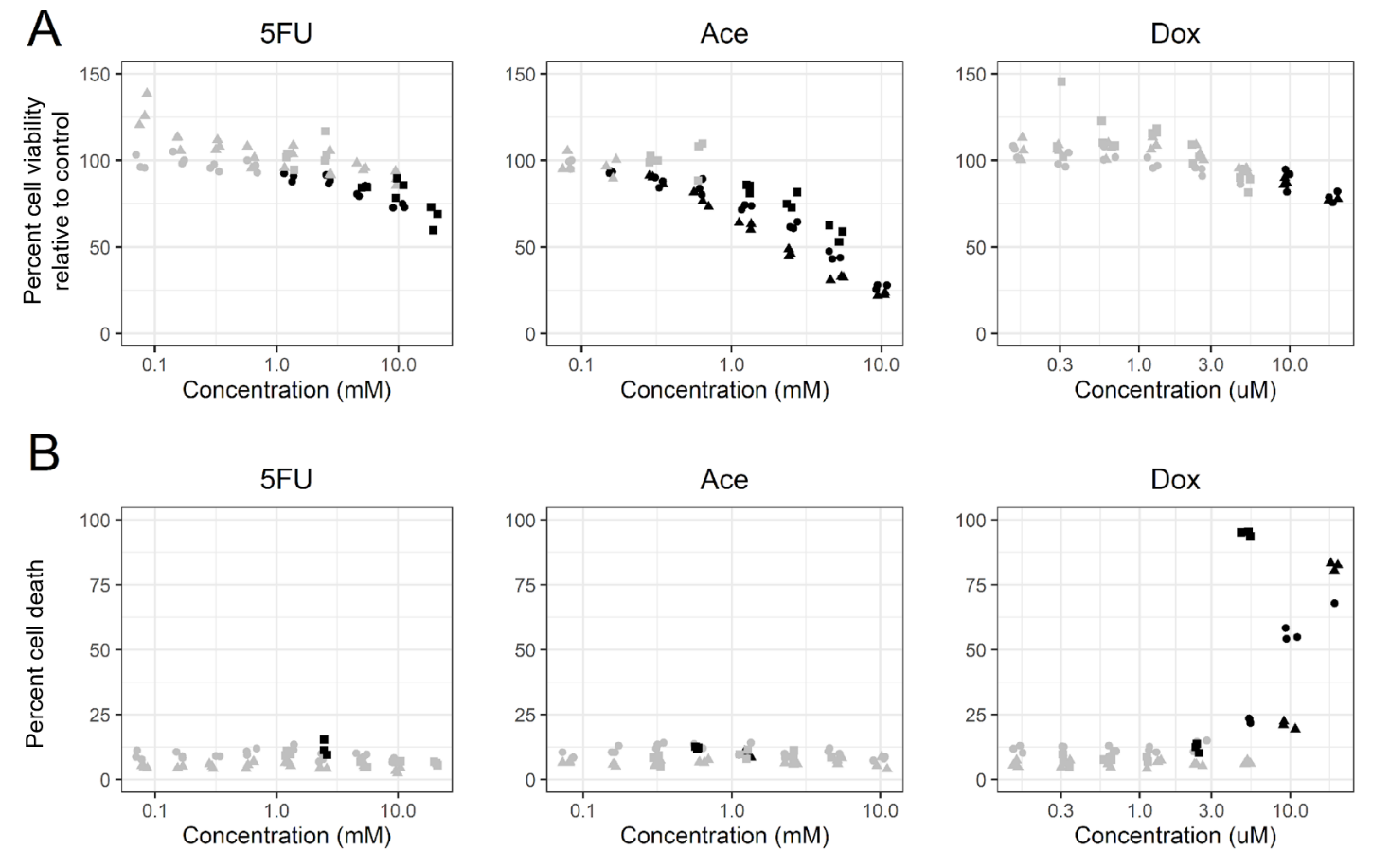

Supplement: S1 Fig — Shapes indicate different cell isolations. Black dots indicate a statistically significant change from the control condition (p-value < 0.05) calculated using Dunntt’s test. Black boxes indicate the chosen concentrations for cardiotoxicity characterization. (A) Percent cell viability for a range of concentrations of treatment following 6 hours of exposure. (B) Percent cell death measured using a Hoescht/PI stain. (TIF) [file pcbi.1011919.s001.tif]

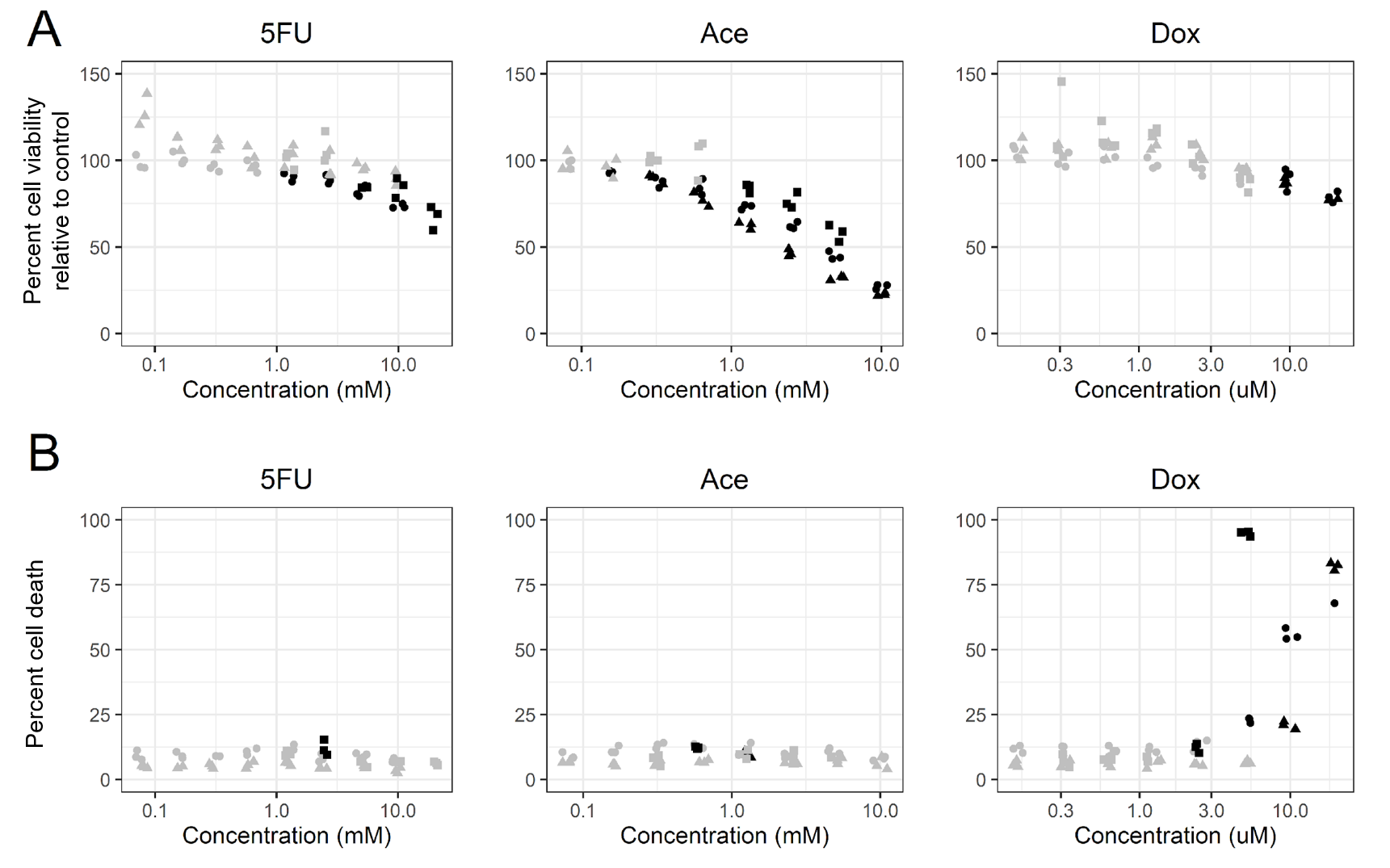

Supplement: S2 Fig — (A) PCA with the Dox samples removed demonstrates separation between treated and control samples at both 6 and 24 hours. (B) The top 10 metabolites show separation in both the first and second principal component. For the first principal component, ethylmalonate and erythritol have a strong influence, suggesting a phenotypic switch between fatty acid and glucose utilization over time, although the direction is unclear. For the second principal component, phosphate and uracil separate the 5FU condition. (TIF) [file pcbi.1011919.s002.tif]

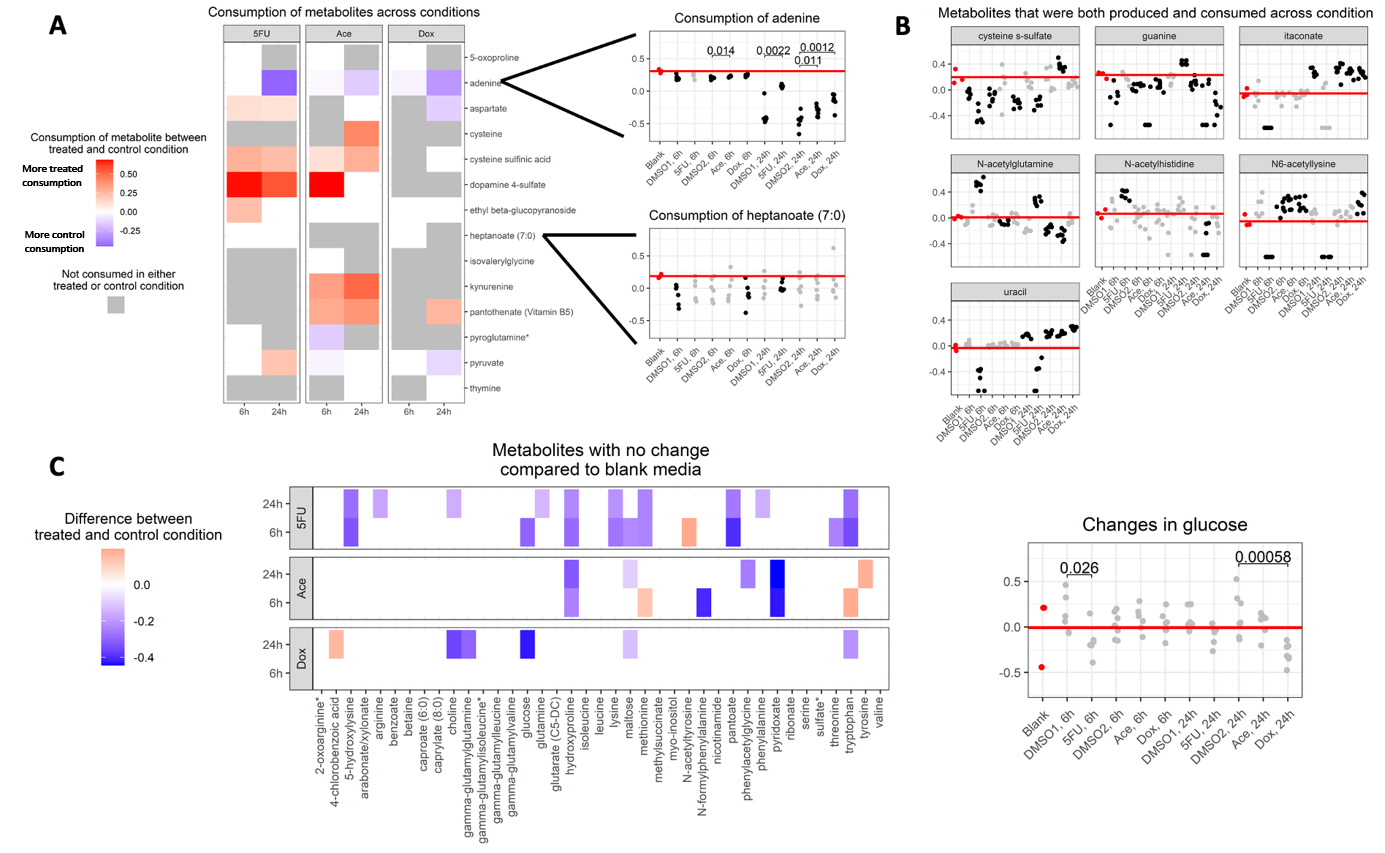

Supplement: S3 Fig — (A) Metabolites that were measured to only be consumed across both drug treated and control conditions when compared to blank media. Here, red indicates a metabolite that was consumed more in the treatment condition than the control condition, blue indicates a metabolite that was consumed more in the control condition than the treatment condition, white indicates a metabolite that was consumed in either/both the treatment and control conditions but there was no difference in consumption and grey indicates a metabolite that was not consumed in either the treatment or control condition when compared to blank media. An example is shown for a metabolite that was more consumed between treated and control conditions (adenine) and a metabolite (hepatonate) that was consumed in a treated/control condition but there was no difference between the treated/control conditions. Black dots indicate a condition where there was a significant change in the metabolite when compared with blank media (Mann Whitney U-test, FDR < 0.1). P-values are shown for comparisons that were significant between a treatment and its respective control. (B) Metabolites that were measured to be both produced and consumed across conditions. Here, the red line indicates the mean value for the blank media samples. Black dots indicate conditions where there was a significant change when compared to blank media (Mann Whitney U-test, FDR < 0.1). A black dot above the red line indicates that a metabolite was produced whereas a black dot below the black line indicates a metabolite that was consumed. (C) Metabolites that were not significantly changed from blank media samples but were measured to change between treatment and control samples. Here, red indicates that a metabolite was present at a higher level in the treated group compared to the control group whereas blue indicates a metabolite that present at a lower level compared to the treated group. Given that there is no significant change with respect to th [file pcbi.1011919.s003.tif]

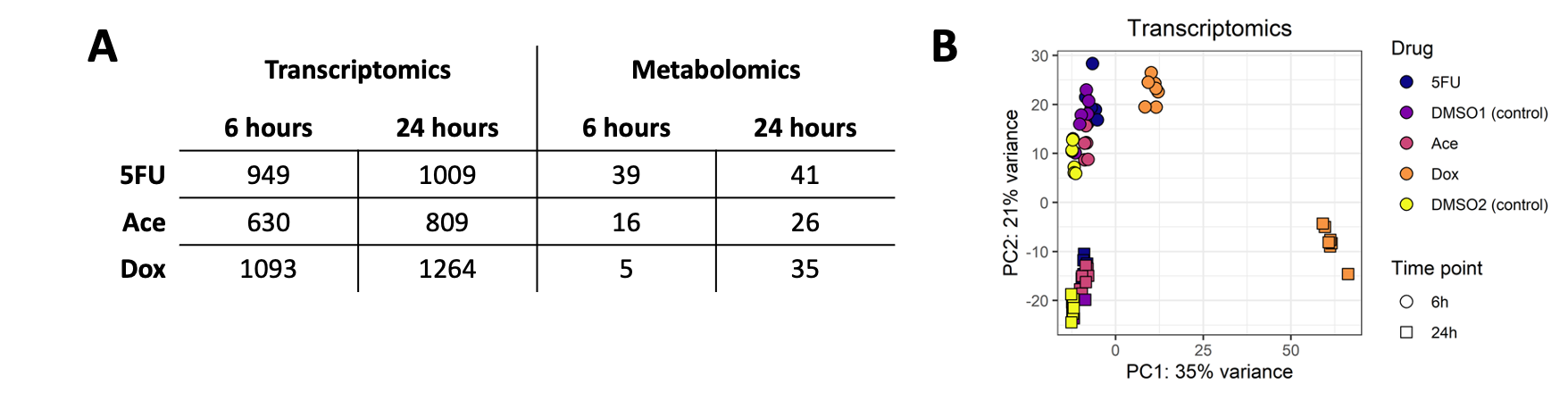

Supplement: S4 Fig — (A) The number of DEGs (FDR < 0.1) and differentially changed metabolites (FDR < 0.1) that map to the rat-specific metabolic mode. (B) A PCA of the normalized gene counts that map back to the rat-specific heart model demonstrate clear separation, similar to the PCA of all gene counts (Fig 2), confirming that metabolism has a large determinant in separating conditions. (TIF) [file pcbi.1011919.s004.tif]

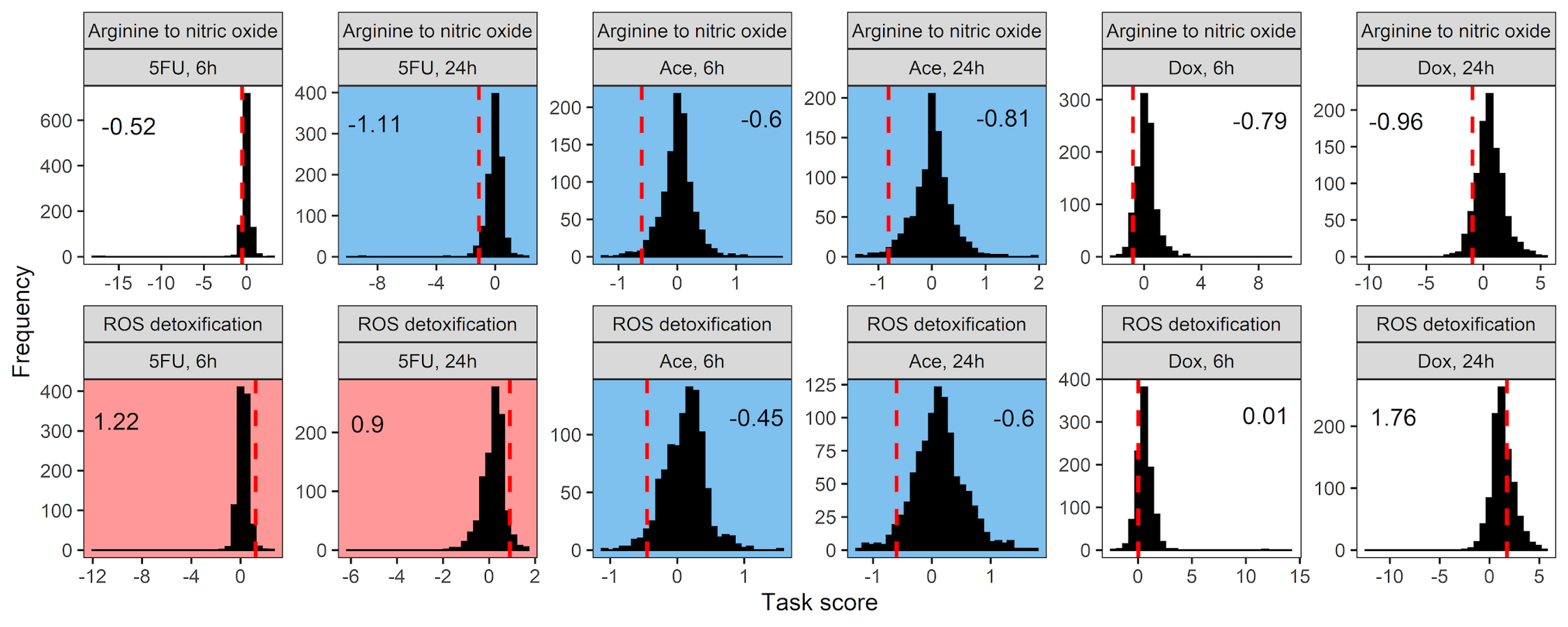

Supplement: S5 Fig — A red background indicates a metabolic task associated with a significant increase in gene expression and a blue background indicates a metabolic task associated with a significant decrease in gene expression (p-value < 0.1). The red dashed line indicates the task score for the actual gene expression data whereas the black bars indicate the calculated task scores when the gene expression data is randomized. In this case, the distribution for the Dox data is significantly wider (i.e. larger range on the x-axis), indicating a larger overall absolute change in gene expression requiring a higher overall average gene expression for ROS production to be deemed significant and a lower overall average gene expression for arginine to nitric oxide to be deemed significant. (TIF) [file pcbi.1011919.s005.tif]

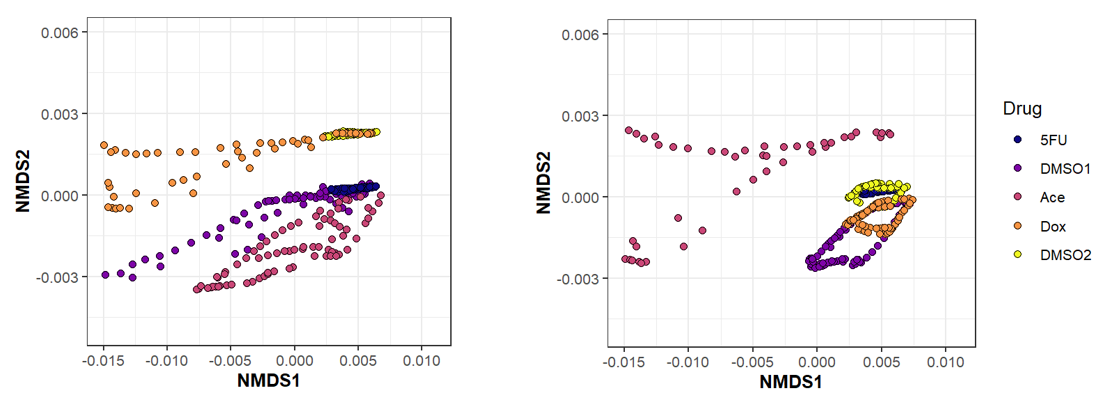

Supplement: S6 Fig — NMDS of the 50 flux samples for each condition at (a) 6 hours and (b) 24 hours. For each flux sample, fluxes were only taken for the 112 reactions that are shared between all conditions. (TIF) [file pcbi.1011919.s006.tif]
