# Supplementary material for: Identifying metabolic adaptations characteristic of cardiotoxicity using paired transcriptomics and metabolomics data integrated with a computational model of heart metabolism
Source: PLoS Comput Biol. 2024 Feb 29;20(2):e1011919. doi: 10.1371/journal.pcbi.1011919 (PMC10931521; doi:10.1371/journal.pcbi.1011919)
Supplement: Attachment — Submitted filename: Response to Reviewers 1.1.docx [file pcbi.1011919.s011.docx]

We thank the reviewers for their comments and insight. Their contributions have greatly improved the quality of the manuscript. The original text of the reviews are shown in **black**, and our response to each comment is shown in **blue**.

—----------------------------

Reviewer #1: This manuscript deals with the survey of the transcriptomics and metabolomics changes occurring in primary cultures of neonatal rat cardiomyocytes exposed to the action of common oncologic drugs, namely 5-fluorouracil, acetaminophen, and doxorubicin. A network metabolic model was reconstructed from the gene expression data based on the human cardiomyocyte model and combined with the multi-omics data to gain mechanistic insights into the effects of and adaptations to these chemotherapeutic agents that lead to cardiotoxicity.
I have a series of comments and criticism that the authors should address. The present version of the manuscript is not satisfactory or appropriate for the broad readership of PLoS Computational Biology.

We thank the reviewer for their helpful comments. We have made efforts to clarify the results of our work and added information that will be helpful for the reader to understand the conclusions of these analyses. We added several points meant to more clearly explain the limitations of our model, the methodology of our work, and the interpretation of our figures.

Lines 99-100. The authors should discuss how these concentrations compare to those achieved in serum during chemotherapy of cancer patients to evaluate whether the toxicity levels achieved in the cultures may occur in vivo during treatment.

We added this information to the discussion.

Line 102. Does the denomination “cell reducing potential” refers to a property related to the redox potential? Because of the importance of the results the authors should specify how is “cell reduction potential” defined and how it is experimentally determined. A reference to a product kit is not enough.

We clarified how reducing potential was measured in the Methods section.

Lines 178-185. The list of DEG that you are highlighting should be included at least as a supplementary table.

A list of DEGs and their related statistics can now be found on the GitHub page.

Line 187. Phosphate. Do you mean free phosphate or phosphorylated metabolites? Please clarify.

We have clarified this point in the manuscript.

Lines 186-207 Regarding the bioinformatic analysis, the authors do not take full advantage of the “multi-omics” approach. An integrated multi-omics analysis of metabolomics-transcriptomics can be performed correlating observed changes with the purpose of determining which pathways share both significant transcripts and metabolites.

You are absolutely correct; however, the scope of this paper focuses on the connections between these data and the rat-specific metabolic model. We have added additional references citing how these data can be further utilized in a more comprehensive “multi-omics” manner in the discussion.

Lines 223-224. Do the 121 metabolites include those with changed abundance (as in table 2B) in response to the treatments with the oncologic drugs?

These metabolites do include those which have changed abundance, and their overlap is summarized in Supplementary Figure 5. We have added this information to the manuscript.

Lines 233-234. This is a real limitation since the GEMRE model ignores 90% of the DEGs that may be the ones providing important mechanistic information about the drug action and cellular adaptive responses that could deliver hints to avoid the cytotoxicity. This should be discussed in a limitations section.

This is an important caveat of our analyses and has been added to the discussion.

Lines 257-262. With so many acronyms those sentences are extremely difficult to understand except for experts in GENRE and TIDE approaches. The authors should include a table with acronyms which are overabundant in the manuscript. When approaches are combined, the authors should refer to the biochemical or conceptual basis thereof instead of assuming the readers’ familiarity with specific jargon.

We have decreased the jargon and clarified the methods in this section.

Lines 293-294. Unclear sentence please rephrase.

We clarified this sentence in the manuscript.

Line 239. Despite the name of the enzyme catalyzing the reaction, PEP under physiological conditions is never directly obtained from Pyruvate. Pyruvate kinase (PK, all isoforms) behaves as irreversible and in the boundary conditions of the model it should be stated as such. In those organs performing gluconeogenesis there is a carboxylation of pyruvate to oxalacetate followed by the PEPCK catalyzed reaction to circumvent the lack of reversibility of PK. Although adult hearts are devoid of gluconeogenic enzymes, their presence may be possible in neonatal cardiomyocytes. The authors should check this possibility and correct the model and Figure 5A.

We have found support for gluconeogenesis occurring in proliferating neonatal cardiomyocytes and included this information in the discussion with relevant citations. Regarding the comment on the reversibility of PK, we have found database references (KEGG: R00200, BRENDA: EC 2.7.1.40, Metabolic Atlas: MAR006627) that suggest this reaction may be reversible. We certainly welcome any citations that PK is not reversible in neonatal cardiomyocytes…to the points the reviewer brings up… so that we can correct the model as needed.

Lines 315-316. The choice of ATP hydrolysis as objective function appears somewhat arbitrary. I believe this issue deserves a more elaborated discussion, including the dismissal of other possible functions in a limitations section.

We have added a section highlighting this point in the discussion.

Figures in the supplement are very confusing:
Supplemental Figure 1 shows that the cells do not produce ATP unless stressed by the oncologic drugs, since DMSO shows negligible ATP production flux as measured by the Seahorse stress test. This result is rather counterintuitive which means that the cardiomyocytes under normal culture conditions display the same basal and oligomycin sensitive respiratory flux. And only the intoxication of the cultured cells with the drugs stimulates the cells to synthesize ATP under culture conditions. This should be explained and interpreted. Perhaps the OCR tests can be repeated in the presence of a beta-adrenergic challenge.

Looking at our raw data, our DMSO group has a comparable decrease in OCR in response to oligomycin which indicates a measure of ATP production. However, there are more live cells in the DMSO condition, so when normalized by this metric, it appears that there is little ATP production compared to the treatments which kill more cells. Additionally, the addition of just DMSO has been shown in the past to ATP production. We have edited the results and discussion sections to clarify this point.

Supplemental Figure 4 I believe the meaning of consumption of metabolites should be clarified. What does it mean to have a negative consumption? It would be much easier to interpret if the authors present the relative abundance and when describing the results mention that a negative change would correspond to consumption and positive to accumulation or production.

This figure was meant to show a comparison of consumptions between control and treatment conditions. We have added additional information to the figure and reworded the figure’s caption to more clearly describe this idea.

Reviewer #2: In their manuscript Dougherty et al. combine transcriptomics and metabolomics data on the same samples in order to study drug-induced in vitro cardiotoxicity with three commonly prescribed cancer therapies. Additionally, they use the data along with a genome-scale metabolic model in order to reveal mechanistic processes underlying drug-induced cardiotoxicity. They identified DEGs and differentially changed metabolites at two time points by comparing treated vs. DMSO control rat cardiomyocyte samples. They apply gene enrichment analysis and discuss recovered pathways in the context of toxicity. Next they build a rat heart GENRE by combining published models including their previously published iCardio model and identified known metabolic tasks as well as potential novel ones.

The manuscript describes an interesting combination of several omics data and in particular how transcriptomics data can be combined with a metabolic model in order to reveal changes in the metabolism. It describes a data analysis work flow, no novel algorithms are developed.

We thank the reviewer for their constructive comments. We focused on clarifying the novel results of our study and the significance of these conclusions. We also make improvements to our analysis and more clearly explain the methods used for these analyses.

My comments are:

1. Novelty: The difference to their previous paper seems to be mainly in the application of their iCardio model (adapted to the rat) with novel drug treatment data. This could be interesting from a toxicogenomics aspect but I do not see much algorithmic or methods development. Please clarify.

We believe the creation of a new rat-specific heart GENRE, the findings on potential mechanisms of toxicity, and the generation of new datasets for use by other researchers are the novel contributions of our paper. We have edited the manuscript to highlight these points to better clarify this novelty.

2. Significance: Authors should better highlight what the novel insights of the approach and the potential for users in the field are. There are multiple transcriptomics studies on Dox that also report metabolic changes and identify – amongst others – altered metabolic processes in the context of oxphos or others. What are the novel findings? Please compare to some of these and show that the used approach is more powerful.

We have edited the manuscript to more clearly outline the significance of our current approach. We compare both TIDEs and RIPTiDe to a commonly used “enricher” method and more explicitly describe how these methods provide more functional information about changes in metabolism. We also discuss in a limitation paragraph other available methods which may provide additional insight not seen in this paper.

3. DMSO controls: DMSO has been shown to induce broad effects in the methylome of cells (e.g. PMID: 30874586) which in fact can exceed the effects of the treatments under study. How is this reflected by the different dosages for DMSO1 and DMSO2? I would suggest computing DEGs from these two controls and test what TIDEs and pathways are found by these DEGs. This would help in evaluating the results.

We have made additions to Figure 2 to include this comparison and discussed its relevance in our limitation section of the discussion.

4. Data normalization: How was transcriptome and metabolome data normalized? This is a crucial point and it should be described in detail in the Methods section.

Data normalization procedures are now in the Methods section.

5. Differential expression (Methods, Figure 2C, page, 20ff): It would be good to have numbers for up- and down-regulated genes. Additionally, are all DEGs protein coding? The RNA protocol is based on poly-A, so I assume read outs also containing long non-coding RNAs and pseudogenes for example. Since the main interest here is on enzymes and metabolically relevant protein coding genes, I would suggest giving the respective numbers for this category.

We have re-counted our DEGs with only protein-coding genes and updated Figure 2C. We also included a supplementary table including up- and down-regulated genes.

6. Pathway enrichment analysis (page 7 and 21): Please define pathway enrichment in the Methods section. Since only DEGs are used for pathway enrichment, I assume authors use exact Fisher test? In the light of the comment above, please reduce DEGs to protein coding genes otherwise the statistics might be misleading.

For this analysis, we use the “enricher” function from the “clusterProfiler” package in R. This method uses a hypergeometric test to determine significance, and it removes any genes without a functional annotation before analysis. We have added information to the Methods section to clarify this point.

7. Sarcomere effects: I wonder a bit that pathway enrichment gives so little information on sarcomere effects. For example, there are strong influences of Dox for muscle contraction and in fact clinical markers of cardiomyopathy, such as TNNT2, NEBL etc., are related to Dox toxic effects. It is a bit puzzling that no cardiomypathy disease gene set is fished by the enrichment. Or may be these are not included in the "hallmarks signatures". Please explain?

This group of “hallmark signatures” does not include any cardiomyopathy-related gene sets. An analysis comparing pathology gene sets could be an interesting analysis for future work.

8. There are several tools that provide common enrichment analysis for genes and metabolites, for example the Impala web server (PMID: 21893519). It would be interesting to compare the analysis to the output of such an analysis.

We agree that additional analyses like the one you suggested would provide valuable support for our findings in this manuscript, but we feel they fall outside of the scope of the goal of this paper. We have added a section in the manuscript mentioning this shortcoming and suggest further work in this regard.

9. Page 6, line 131: enrichment was computed for the top 100 genes; this is quite an arbitrary threshold. Please motivate.

We used this threshold as it had been used previously with success (Subramanian et al., 2005). We cite this paper in the Methods section to clarify this point.

10. Page 7, line 155: top 10 metabolites; same comment.

You are correct that this cutoff is arbitrary; however, since these top 10 metabolites are not used for a further analysis (such as the enrichment with the transcription data) we feel this is an appropriate threshold. We include PC weights for all metabolites in Supplementary File 4 to ensure full transparency.

11. Page 6, line154: „]“ is missing.

We have edited the manuscript to fix this error.

Reviewer #3: The authors present paired transcriptomics and metabolomics data characterizing in vitro cardiotoxicity to three compounds: 5-fluorouracil, acetaminophen, and doxorubicin. The authors methodology identified metabolic adaptations in response to cardiotoxicity vis a vis standard gene enrichment and metabolomics approaches identify some commonly affected pathways and metabolites. The authors claim that shifted metabolic functions, unique metabolic reactions, and changes in flux in metabolic reactions and hence potential mechanisms for metabolic adaptation in response to drugs were identified by integrating the paired data with a genome-scale metabolic network reconstruction of the heart.

In general, the manuscript is poorly written with respect to the results not being very clearly and sufficiently presented. The discussion section is too verbose. All in all, the methods and results are novel and exciting enough to spark excitement, but require more than superficial mention.
At this point a recommend a major revision.

I have a few suggestions:

We thank the reviewer for their helpful comments. We have edited the manuscript to more clearly tie together our results and more effectively discuss the impact of those results. We added information to help the reader more easily understand our work by more explicitly synthesizing our results.

1) It would be nice if in the abstract the authors include some key results from the used integrated approach with respect to both the validation of legacy metabolic adaptations in 5FU and Dox cardiotoxicity, the main new metabolic adaptations in Ace cardiotoxicity and the proposed shifts in key metabolic pathways that show increased metabolic demand in 5FU and Dox elucidating primary chemotherapeutic mechanisms of action.

We have added this information to the abstract to make it more informative.

2) Although the authors mention a significant increase in OCR for ATP production for 10 mM 5FU and 1.25 μM Dox and refer the reader to the supplemental data figure, it would be beneficial to mention a fold change right here.

We have added this information to the manuscript.

3) The authors claim that the only A gene enrichment analysis of the top 100 genes identified p53 pathway as the only significantly enriched pathway in DOX treated cells. Also, they identify ethylmalonate, a branched chain fatty acid, for primary separation in the first principal component, suggesting a change in glucose and fatty when cells are treated with 5FU. However none of these observations are clearly tied up through gene expression/metabolomic data analysis.

We have tied these observations together with our TIDEs results, recognizing agreements between these analyses.

4) Although the Dox condition has the largest number of DEGs at both 6 and 24-hours while the 5FU condition has the largest number of differentially changed metabolites at the 6 and 24-hour conditions, there absolutely no discussion of these results in the text.

We agree that this is an important caveat to mention, and we have added a section in the discussion highlighting the limitation of the link between transcriptomics and metabolomics.

5) It is alsocunclear, how do the authors deduce the consistent increase in production of 2’-deoxyinosine 2’-deoxyuridine in the both the 5FU and Dox treated cells as a marker for ROS.

We only suggest that the increase in 2’-deoxyinosine production suggests the presence of ROS citing the literature as evidence (Lee et al., 2015; citation [36] in the manuscript). 2’-deoxyuridine only shows changes in DNA damage and uracil metabolism. We have made this point clearer in the manuscript.

6) Although uracil was identified as differentially utilized or produced under the varying
treatment conditions at 24 hours, again there is no correlation made to DEGs.

We have now directly linked our TIDEs analysis with DEGs to our metabolomics results in the manuscript.

7) The model captures ~10% of the DEGs across all treatments and time points and between 40-65% of the differentially changed metabolites. It would be useful to mention some key metabolites. The a shift from glycolytic metabolism to fatty acid metabolism as a source of ATP is not supported by data that was collected from the experiments.

We have now explicitly mentioned pyruvate and glucose as key changed metabolites that are in the model.

It is true that the direction of this shift is not supported; however, we do observe changes in both pathways through the enrichment and PCA analyses. Consequently, we have softened these claims and more clearly linked this conclusion to the data.

8) There are many typographical and grammatical errors and words in between sentences suddenly start with a capital letter.

We have edited the manuscript to fix these errors.
