# Supplementary material for: Identifying metabolic adaptations characteristic of cardiotoxicity using paired transcriptomics and metabolomics data integrated with a computational model of heart metabolism
Source: PLoS Comput Biol. 2024 Feb 29;20(2):e1011919. doi: 10.1371/journal.pcbi.1011919 (PMC10931521; doi:10.1371/journal.pcbi.1011919)
Supplement: Attachment — Submitted filename: Response to Reviews - Identifying metabolic adaptations 2.1.docx [file pcbi.1011919.s012.docx]

We thank the reviewers for their comments and insight. Their contributions have greatly improved the quality of the manuscript. The original text of the reviews are shown in **black**, and our response to each comment is shown in **blue**.

—----------------------------

Reviewer #1: The new version of the manuscript by Dougherty and coworkers does not show a significant improvement over the original one. I would have expected that after six months the authors would have incorporated a more elaborate research work; instead, I found in their revised manuscript just some edits, rewritten text, and clarifications, at least concerning the points I originally raised.

We appreciate the critiques to date and feel the manuscript continues to improve. We have addressed the additional points below.

Regarding the issue of the reversibility of the pyruvate kinase (PK), the authors have not addressed my concerns in the revised manuscript. The PK may under controlled circumstances (in vitro) operate in the reverse direction. However, as I said in my original report, the chances of this happening under physiological conditions are slim. The PK affinity constant for Pyruvate is in the order of 7 mM when actual levels of the metabolite in vivo in most mammalian cells rarely reaches 100 microM, making the reverse reaction rather unlikely or extremely slow. The evolutionary selection of the activities of pyruvate carboxylase and PEPCK highly expressed in gluconeogenic organs such as liver and kidney, confirm the need of an alternative path to overcome the slow or negligible reverse flow through PK. I was expecting to find the results with a model incorporating these activities in their formulation, but the authors did not even attempt to perform such a small but significant change in their modeling.

We understand the reviewer’s critique. We redid our analysis with this additional constraint (setting PK as irreversible) and updated our findings accordingly (Lines 334-356). Though it affects the specific reaction we highlighted in the previous manuscript, our results still suggest a change in pyrimidine metabolism in response to treatment as previously highlighted.

Regarding the very low rates of ATP production in the case of the control due to larger number of viable cells in the respiration assay is an indication of the lack of linearity of the assay with respect to the number of cells in the well. This is an essential experimental condition that should be optimized before any assay in this system can be performed, Otherwise, the assay is saturated in the wells with many cells (e.g., those with the control cells in the absence of cardiotoxic drugs) and is likely still in the linear range for the small number of cells in the “treated” wells. Certainly, the comparisons should be made between wells with similar numbers of functional cells, to avoid issues of saturation of the signal. Given the lack of linearity in the assay, the comparison between control and treated cells is meaningless, and those results should be discarded.

In evaluating these data to which the reviewer is referring, we realized they did not greatly affect our conclusions, and we have removed these data from the Supplemental Materials, Results, and Methods sections of this manuscript.

The answers to my original comments lack details e.g., about where to find the modified parts in the revised manuscript. In one case, the authors mentioned the list of Differentially expressed genes (DEG) in a website in GitHub in a directory where there are a large number of files, without even mentioning the file name of the document containing the requested information or including such information in the revised manuscript.

We more clearly organized descriptions and data on the DEGs (found at <https://github.com/BonnieDougherty/Cardiotoxicity/tree/main/data/RNA-seq/DEGs>) and mentioned this update in the Data Availability section of the manuscript.

Concerning the comment with respect to the “cell reducing potential” I insist that the denomination is confusing, and, in that context, should be replaced by “cell viability”. However, the reagent used has been in the market for relatively few years and there are no reports about how senescent cells (that have been demonstrated to be generated by oncologic drugs, e.g. Prasanna et al, 2021 PMID: 33792717) will behave in terms of reducing the commercial reagent used to assess “viability”. Thus, I have strong reservations regarding the interpretation of those results.

We have clarified this point in the manuscript (Line 501) by acknowledging the reagent’s stated use to measure “cell reducing potential” on Promega’s RealTime-Glo™ MT Cell Viability Assay webpage as a measure of cell viability which we now use through the manuscript. We have also added a source (Line 503) showcasing this reagent’s use in several types of cells (<https://doi.org/10.1089/adt.2015.669>).

The limitations section in the discussion is written in a rather defensive way than in acknowledgement of the real limitations of the conclusions reached. For example, when the authors state: “by removing 90% of DEGs from our analysis, we focus on only metabolically relevant genes” without any functional demonstration about the relevance of the included (~10%) or excluded (~90%) genes.

We appreciate this comment and we have revised the discussion (see lines 450-464) to more deliberately acknowledge the limitations of the conclusions reached.

Reviewer #2: The authors have addressed my points sufficiently. The improved ms is suited for publication.

We appreciate the positive evaluation and we are grateful for the feedback during this review process!

Reviewer #3: The authors have addressed all queries posed in the first review. They have included required sections in the text.
The article is now more cogent than the first version.
I thus recommend this article for publication.

We appreciate the positive evaluation and we are grateful for the feedback during this review process!
